# Composition and Orientation of the Core Region of Novel HIV-1 Entry Inhibitors Influences Metabolic Stability

**DOI:** 10.3390/molecules25061430

**Published:** 2020-03-21

**Authors:** Rama Karadsheh, Megan E. Meuser, Simon Cocklin

**Affiliations:** Department of Biochemistry & Molecular Biology, Drexel University College of Medicine, Rooms 10307, 10309, and 10315, 245 North 15th Street, Philadelphia, PA 19102, USA; rk885@drexel.edu (R.K.); mem484@drexel.edu (M.E.M.)

**Keywords:** HIV-1 entry inhibitor, metabolic stability, docking, antiviral, surface plasmon resonance, Cyp P450

## Abstract

Fostemsavir/temsavir is an investigational HIV-1 entry inhibitor currently in late-stage clinical trials. Although it holds promise to be a first-in-class Env-targeted entry inhibitor for the clinic, issues with bioavailability relegate its use to salvage therapies only. As such, the development of a small molecule HIV-1 entry inhibitor that can be used in standard combination antiretroviral therapy (cART) remains a longstanding goal for the field. We previously demonstrated the ability of extending the chemotypes available to this class of inhibitor as the first step towards this overarching goal. In addition to poor solubility, metabolic stability is a crucial determinant of bioavailability. Therefore, in this short communication, we assess the metabolic stabilities of five of our novel chemotype entry inhibitors. We found that changing the piperazine core region of temsavir alters the stability of the compound in human liver microsome assays. Moreover, we identified an entry inhibitor with more than twice the metabolic stability of temsavir and demonstrated that the orientation of the core replacement is critical for this increase. This work further demonstrates the feasibility of our long-term goal—to design an entry inhibitor with improved drug-like qualities—and warrants expanded studies to achieve this.

## 1. Introduction

The HIV-1 Env glycoprotein complex on the surface of a virion orchestrates the two sequential and specific binding events that allow for entry into susceptible cells. Env is a heterotrimer of gp41 and gp120 subunits that undergo the conformational changes, prompted by receptor engagement, necessary for the fusion of viral and cellular membranes. As Env is the primary determinant of viral infectivity, it is an attractive target for both pre- and post-exposure prophylaxis and is the focal immunogen of the search for a viable vaccine. Two entry inhibitors are in use in the clinic (enfuvirtide and maraviroc) but are only suitable for salvage therapies. Thus, a well-tolerated, orally bioavailable, and virally-targeted entry inhibitor that can treat the majority of isolates of the virus has been actively sought for many years.

The most promising entry inhibitor class has been developed by Bristol-Myers Squibb. The initial compound, BMS-378806, and subsequent iterations, share a piperazine core chemotype and target the HIV-1 Env gp120 subunit [1]. The most promising analog from this class of inhibitors, BMS-626529 ((ViiV Healthcare: Temsavir), is potent against many of the major subtypes of HIV-1, but unfortunately suffers from poor bioavailability due to its low solubility. However, BMS-663068 (ViiV Healthcare: Fostemsavir), a phosphooxymethyl prodrug of temsavir, has performed favorably in clinical trials and is slated for release late 2020 [2]. Unfortunately, due to suboptimal solubility after cleavage of the prodrug, and breadth issues against specific subtypes of HIV-1, this entry inhibitor is only recommended for treatment-experienced patients with limited therapeutic options. 

Our group has been actively exploring the bioisosteric modification of the piperazine-based entry inhibitors, as exemplified by temsavir/fostemsavir. This is part of an overarching effort to improve upon their drug-like properties and breadth and to hopefully bring them closer to use in standard combination antiretroviral therapy (cART) [3,4,5]. We have performed proof-of-concept studies demonstrating that we can scaffold-hop from the piperazine chemotype while retaining nanomolar inhibitory profiles and retaining target engagement. However, before this study, we were yet to investigate whether these initial new chemotypes have any improved drug-like qualities. Therefore, in this short communication, we investigate whether any of our novel chemotypes have any improved ADME (absorption, distribution, metabolism, and excretion) properties using both computational and experimental methods. Moreover, in the course of this study, we establish a computational workflow that can be used to predict the metabolic stability of next-gen compounds, thus further streamlining future design processes. 

## 2. Results and Discussion

**Figure 1:**
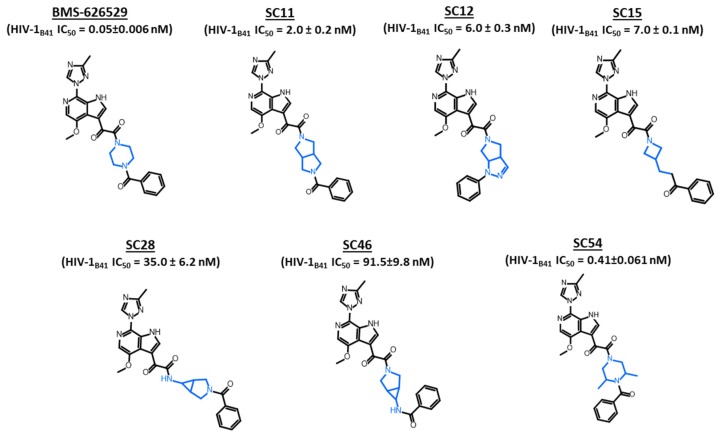
Chemical structures of the compounds used in this study. Half maximal inhibitory concentrations (IC_50_) against HIV-1_B41_ Env pseudotyped HIV-1 are given. Structural differences in the core regions are highlighted in blue. The chemical structures were drawn with ChemAxon software (Budapest, Hungary).

We have previously successfully used field-based three-dimensional similarity virtual screening experiments, in combination with bioisosteric replacements, to identify novel scaffolds that could function as entry inhibitors [3,4]. These studies resulted in the discovery of compounds **SC11** (dipyrrolidine), **SC12** (pyrrolo-pyrazole), **SC15** (azetidine), and **SC28** (azabicyclo-hexane). These compounds display IC_50_ values in the 2–40 nM range against HIV-1_B41_ Env pseudotyped HIV-1 (Figure 1). In this study, we introduce two additional compounds, **SC46** and **SC54**, which have a reversed azabicyclo-hexane and a dimethyl-piperazine core, respectively. These new compounds also have inhibitory effects on HIV-1_B41_ Env pseudotyped HIV-1 in the low nanomolar range (Figure 1). 

**Figure 2:**
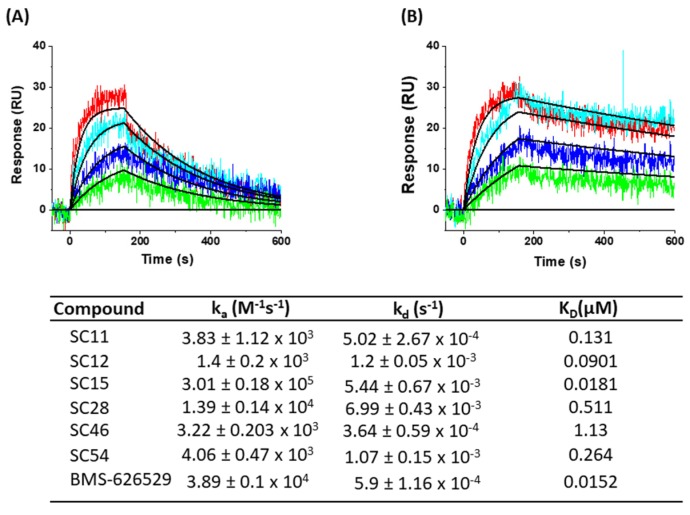
Sensorgrams depicting the interaction of (**A**) SC46 and (**B**) SC54 with recombinant trimeric HIV-1_B41_ SOSIP Env. Colored lines represent the raw data of the different concentrations of compound used, and the black lines represent the fit that was derived using a 1:1 binding model. A table showing the interaction parameters for all compounds used in this study is also provided.

Moreover, all of these compounds (**SC11**, **SC12**, **SC15**, **SC28**, **SC46**, and **SC54**), as well as temsavir/BMS-626529, interact with the SOSIP recombinant Env trimer derived from the B41 isolate [6] with different kinetics (Figure 2).

**Figure 3:**
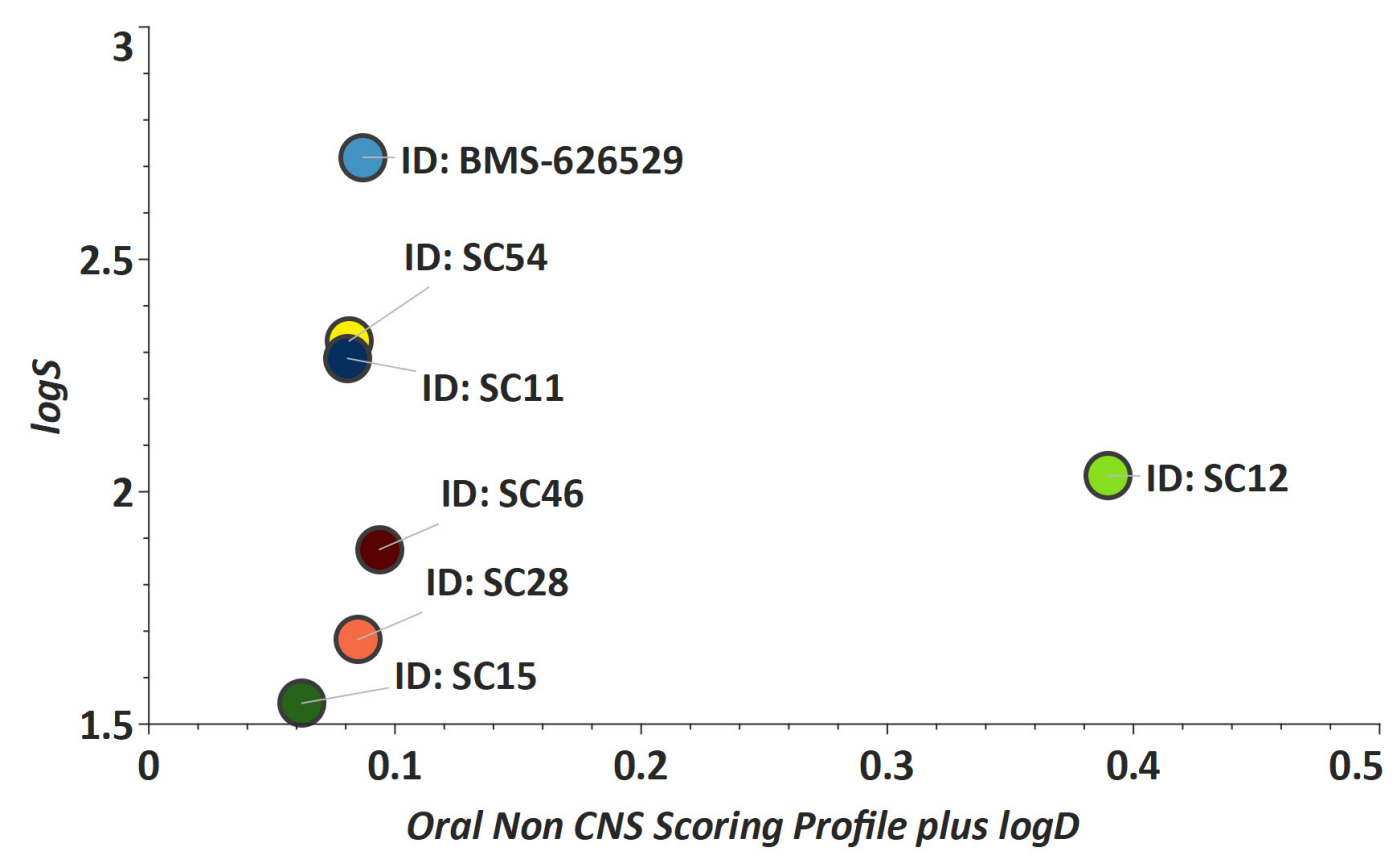
Plot showing the StarDrop 6.6 (Optibrium, Ltd., Cambridge, UK)-derived logS versus the score from a multimetric oral non-CNS profile for the BMS and SC compounds used in this study.

Having demonstrated antiviral activity and target engagement for the previously published and new compounds, we next analyzed the predicted ADME properties of the compounds and compared them with those of the temsavir/BMS-626529. To accomplish this comparison, we performed in silico prediction of drug-like metrics of the compounds as implemented in the oral non-central nervous system (CNS) drug profile in StarDrop 6.6 (Optibrium, Ltd., Cambridge, UK) [7]. We have previously described this drug profile and component models in Tuyishime et al. [3], and additional details are online at the StarDrop FAQs (http://www.optibrium.com/community/faq/adme-qsar-models). A probabilistic scoring algorithm [8] is used to combine all of the component model predictions in the oral non-CNS drug profile into an overall score, with scores range from 0 to 1 (0 suggesting extremely non-drug-like, and 1 indicating the perfect drug). A plot of this metric versus aqueous solubility (logS) is shown in Figure 3. As can be seen, none of the new chemotypes display improved solubility compared to temsavir/BMS-626529. However, **SC12** had an overall improved oral non-CNS drug profile score, primarily due to improved scoring against the human intestinal absorption model.

**Figure 4:**
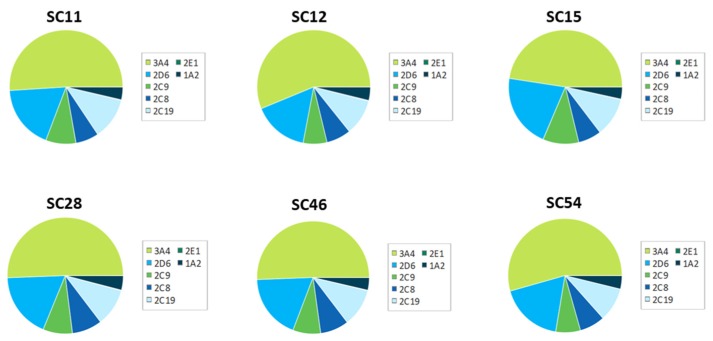
Prediction of the major metabolizing CYP isoform for the compounds in this study. Prediction was achieved using the StarDrop (version 6.6) P450 module. BMS-626529 was also predicted to be predominantly metabolized via the 3A4 isoform.

As the bioavailability of temsavir/BMS-626529 is a primary reason for its use only in salvage therapies and none of our first-generation chemotypes displayed improved predicted aqueous solubility, a primary determinant of bioavailability, we chose to investigate further. Orally administered drugs must pass through the intestinal wall and then the portal circulation to the liver, with both sites being common regions of first-pass metabolism. As such, many drugs may be adversely metabolized before adequate plasma concentrations are reached. Therefore, we next sought to computationally investigate whether or not our compounds had improved predicted metabolic stability. We performed this analysis using the P450 module in StarDrop 6.6 (Optibrium, Ltd., Cambridge, UK). We first predicted the major metabolizing cytochrome P450 isoforms for each compound using the WhichP450™ model [9]. The results of this analysis are shown in Figure 4.

All of the SC compounds are predicted to primarily be metabolized by CYP3A4. Temsavir/BMS-626529 was also predicted to be metabolized by CYP3A4, which has been determined to be the primary metabolizing isoform [10] As such, we next analyzed the predicted metabolic lability of each of the compounds against CYP3A4 by comparing the overall composite site lability (CSL) score and the number of labile sites [11]. The CSL score is a metric that reflects the overall efficiency of metabolism of the molecule and is calculated by combining the labilities of individual sites within the compound. Table 1 shows the CSL scores for CYP3A4 for all of the SC compounds and temsavir/BMS-626529. As can be seen, the differences between the CSL scores of the compounds are minor; there is no significant difference between the numbers of predicted labile sites. Such small changes in the CSL scores are not to be expected to be correlated with appreciable differences in experimental CYP3A4 half-life or intrinsic clearance [11]. This is because other factors that govern the rate of metabolism, such as reduction rates and whether or not the compound binds to the enzyme. Therefore, a primary assumption in this analysis is that all of the compounds bind to CYP3A4 with similar binding affinity, which itself is influenced by compound properties such as size and lipophilicity. We, therefore, performed predictive binding affinity calculations using the hydrogen bond and dehydration (HYDE) energy scoring function in SeeSAR 9.2 (BioSolveIT Gmbh, Germany) [12]. For this analysis, we used the structure of the human CYPA4 bound to an inhibitor (PDB ID 4D78) [13]. The HYDE scoring function in SeeSAR provides a range of affinities, spanning an upper and lower limit. We, therefore, used the lower limit as the affinity parameter to compare between molecules. Table 1 shows the affinity values derived from this analysis. Unlike the previous analyses, the prediction of CYP3A4 binding resulted in more considerable differences between compounds, with the affinities being predicted in the range of 0.8–800 mM. Combining the results from all of these predictions (CSL scores, labile sites, and predicted CYP3A4 affinity), it would appear that compound **SC28** and its core-reversed analog **SC46** should have greater metabolic stability than the other compounds. Additionally, based just upon the number of labile sites, **SC12** should have the lowest metabolic stability.

Armed with our prediction that **SC28** and **SC46** may have increased metabolic stability compared to temsavir/BMS-626529 and the other compounds, we next performed metabolic stability assays using human liver microsomes. Metabolic stability analysis was performed as outlined in Lu et al. [14] and as described explicitly in Materials and Methods. Compounds testosterone (low stability), propranolol (medium stability), and warfarin (high stability) were included as in-line controls. Table 2 shows the metabolic stabilities of the compounds used in this study, along with derived predictive pharmacokinetic (PK) parameters. As can be seen, the compounds have a range of stabilities, as indicated by their respective half-lives (T½). As predicted from the sheer number of labile sites, **SC12** had the shortest half-life of just over 6½ minutes. No clear correlation with any of the other potentially predictive parameters could be gauged for compounds BMS-626529, **SC11**, **SC15**, and **SC54**. However, rather pleasingly, compounds **SC28** and **SC46** had the greatest half-lives of 144.6 and 91.2 min, respectively, demonstrating a clear correlation with their predicted affinity to CYP3A4.

## 3. Materials and Methods

### 3.1. Compounds

BMS-626529 was purchased from MedChemExpress (Monmouth Junction, NJ, USA). **SC11** was synthesized following the procedures described in Tuyishime et al. [3]. **SC12**, **SC15**, and **SC28** were synthesized as described in Tuyishime et al. [4]. Compounds **SC46** and **SC54** were synthesized de novo, as outlined below.

#### 3.1.1. Synthesis of SC46

General procedure for preparation of Compound **2**

**Scheme 1:**
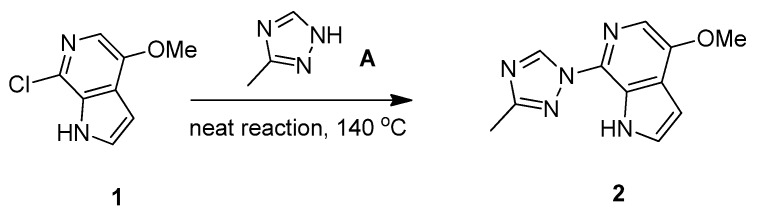
Preparation of Compound **2**.

To a solution of Compound **A** (11.00 g, 132.39 mmol) at 110 °C was added Compound **1** (5.00 g, 27.38 mmol) (Scheme 1). The mixture was stirred at 140 °C for 16 hours under N_2_. TLC (petroleum ether/ethyl acetate = 1:1, Rf = 0.3) indicated ≈30% of Reactant 1 remained, and one major new spot with larger polarity was detected. The residue was purified by column chromatography (SiO_2_, petroleum ether/ethyl acetate = 2/1 to 1:1) to give Compound **2** (3.00 g, 13.09 mmol, 47.80% yield) as a white solid. ^1^H-NMR (ET5676-11-P1B, 400 MHz, CDCl_3_): 10.25 (br. s., 1 H), 9.08 (s, 1 H), 7.56 (s, 1 H), 7.38 (t, *J* = 2.8 Hz, 1 H), 6.71 (t, *J* = 2.6 Hz, 1 H), 4.04 (s, 3 H), 2.54 (s, 3 H).

General Procedure for Preparation of Compound **3**

**Scheme 2:**
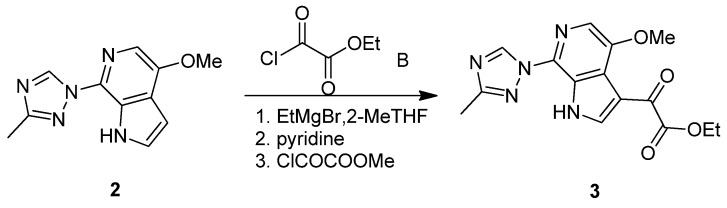
Preparation of Compound **3**.

A mixture of Compound **2** (2.80 g, 12.21 mmol) in 2-MeTHF (120.00 mL) was cooled to −10 °C (Scheme 2). EtMgBr (3 M, 15.02 mL) was added dropwise at −10 °C, followed by the addition of pyridine (500.00 mg, 6.32 mmol). The mixture was cooled to −45 °C, and then Compound **B** (7.00 g, 51.28 mmol) was added. The mixture was stirred at 25 °C for 48 h under N_2_. TLC (petroleum ether/ethyl acetate = 1/1, Rf = 0.2) indicated ≈40% of Reactant 1 remained, and one major new spot with larger polarity was detected. The mixture was quenched with H2O (250 mL), and extracted with ethyl acetate (200 mL × 3). The organic was dried over Na2SO4 and concentrated to give the residue. The residue was purified by column chromatography (SiO_2_, petroleum ether/ethyl acetate = 2:1 to 1:1) to give Compound **3** (2.00 g, 6.07 mmol, 49.74% yield) as a yellow solid. ^1^H-NMR (ET5676-35-P1A, 400 MHz, CDCl_3_): 10.93 (br. s., 1 H), 9.04 (s, 1 H), 8.24–8.31 (m, 1 H), 7.68 (s, 1 H), 4.35 (q, *J* = 7.0 Hz, 2 H), 3.94–4.00 (m, 3 H), 2.49 (s, 3 H), 1.34 (t, *J* = 7.2 Hz, 3 H).

General Procedure for Preparation of Compound **3**-**1**

**Scheme 3:**
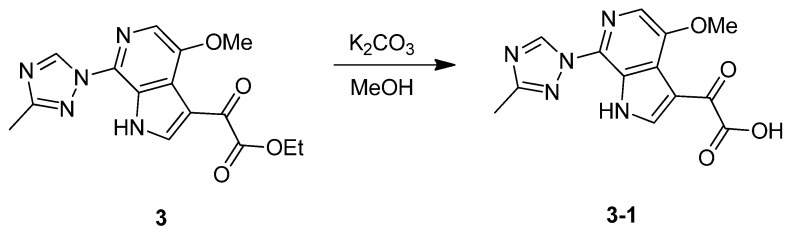
Preparation of Compound **3**-**1**.

A mixture of Compound **3** (1.00 g, 3.04 mmol) K_2_CO_3_ (1 M, 15.34 mL) in MeOH (15.00 mL) was stirred at 20 °C for 1 h (Scheme 3). TLC (petroleum ether/ethyl acetate = 1/1,Rf = 0.01) indicated Reactant 1 was consumed completely and one new spot formed. The reaction was clean, according to TLC. The MeOH was concentrated and diluted with H_2_O (150 mL). The mixture was extracted with ethyl acetate (100 mL × 2). The aqueous phase was acidified with HCl to pH = 1, and the solid was filtered and dried to give a residue. The residue was dried to give Compound **3**-**1** (400.00 mg, crude) as a yellow solid. ^1^H-NMR (ET5676-38-P1B, 400 MHz, DMSO-d_6_): 12.40 (br. s., 1 H), 9.20 (s, 1 H), 8.26 (d, *J* = 3.6 Hz, 1 H), 7.84 (s, 1 H), 3.94 (s, 1 H), 3.13 (s, 1 H).

General Procedure for Preparation of Compound **5**

**Scheme 4:**
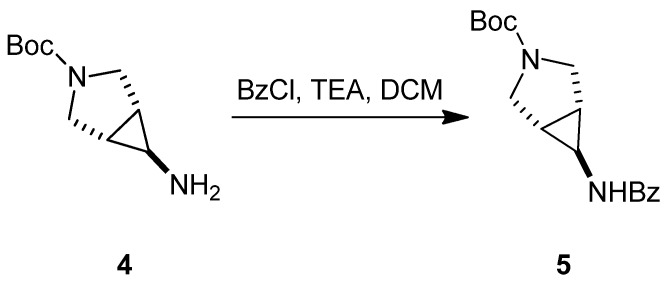
Preparation of Compound **5**.

To a mixture of Compound **4** (1.00 g, 5.04 mmol) and TEA (1.00 g, 9.88 mmol) was added benzoyl chloride (1.00 g, 7.11 mmol) dropwise at 0 °C (Scheme 4). The mixture was stirred at 25 °C for 16 h. TLC (petroleum ether/ethyl acetate = 2/1, Rf = 0.3) indicated Reactant 1 was consumed completely, and one new spot formed. The reaction was clean, according to TLC. The mixture was quenched with H2O (50 mL), and extracted with ethyl acetate (50 Ml × 3). The organic was dried over Na_2_SO_4_ and concentrated to give the residue. The residue was purified by column chromatography (SiO_2_, petroleum ether/ethyl acetate = 2/1 to 1:1) to give Compound **5** (1.40 g, crude) as a white solid. ^1^H-NMR (ET5676-28-P1A, 400 MHz, CDCl_3_): 8.17 (s, 1 H), 7.33–7.39 (m, 2 H), 7.28–7.32 (m, 3 H), 7.20–7.25 (m, 1 H), 6.99 (s, 1 H), 6.95 (s, 1 H), 6.82 (s, 1 H), 6.79 (s, 1 H), 5.42 (s, 2 H), 4.26 (s, 5 H), 3.78 (s, 3 H).

General Procedure for Preparation of Compound **6**

**Scheme 5:**
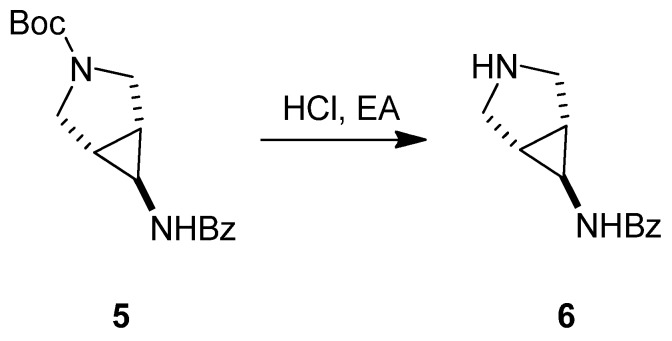
Preparation of Compound **6**.

A solution of Compound **5** (1.40 g, 4.63 mmol) in HCl/EtOAc (30.00 mL, 4M) was stirred at 25 °C for 3 h (Scheme 5). The mixture was concentrated to give Compound **6** (900.00 mg, 3.77 mmol, 81.43% yield, HCl) as a white solid. ^1^H-NMR (ET5676-37-P1A, 400 MHz, DMSO-d_6_): 9.73 (br. s., 1 H), 9.15 (br. s., 1 H), 8.60 (d, *J* = 4.0 Hz, 1 H), 7.80 (d, *J* = 7.2 Hz, 2 H), 7.47–7.54 (m, 1 H), 7.38–7.46 (m, 2 H), 3.25–3.39 (m, 4 H), 2.99 (d, *J* = 2.8 Hz, 1 H), 1.98 (br. s., 2 H).

General Procedure for Preparation of Compound **SC46**

**Scheme 6:**
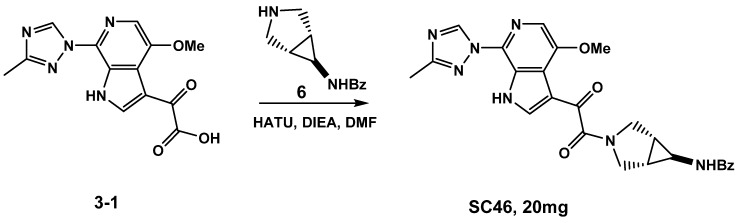
Preparation of Compound **SC46**.

A mixture of Compound **3-1** (700.00 mg, 2.32 mmol), Compound **6** (610.00 mg, 2.56 mmol), DIEA (900.00 mg, 6.96 mmol) and HATU (1.32 g, 3.48 mmol) in DMF (25.00 mL) was stirred at 28 °C for 15 h under N_2_ atmosphere (Scheme 6). LC-MS showed Reactant 1 was consumed completely, and one peak with desired MS was detected. The mixture was poured into H_2_O (150 mL), and extracted with DCM (100 mL × 3). The mixture was dried over Na_2_SO_4_ and concentrated to give a residue. The residue was purified by prep-HPLC (basic condition) to give **SC46** (30.00 mg, 2.65% yield) as a white solid. ^1^H-NMR (ET5676-52-P1B, 400 MHz, DMSO-d_6_): 12.38 (br. s., 1 H), 9.21 (s, 1 H), 8.56 (d, *J* = 4.0 Hz, 1 H), 8.22 (s, 1 H), 7.87 (s, 1 H), 7.79 (d, *J* = 7.2 Hz, 2 H), 7.47–7.53 (m, 1 H), 7.37–7.46 (m, 2 H), 3.99 (s, 3 H), 3.89 (d, *J* = 11.6 Hz, 1 H), 3.63–3.70 (m, 1 H), 3.59 (d, *J* = 10.8 Hz, 2 H), 2.64 (br. s., 1 H), 2.52 (d, *J* = 2.4 Hz, 3 H), 1.92 (br. s., 2 H).

#### 3.1.2. Synthesis of SC54

General Procedure for the Preparation of Compound ***7***

**Scheme 7:**
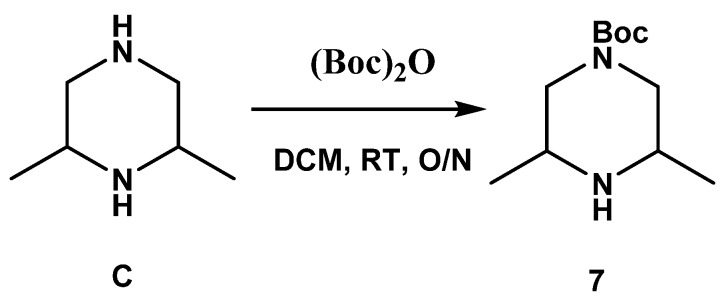
Preparation of Compound **7**.

To a mixture of 2,6-dimethylpiperazine (Compound **C**, 1.0 g, 8.8 mmol) in DCM (10 mL) was added (Boc)_2_O (1.9 g, 8.8 mmol) at RT (Scheme 7). After stirred at RT overnight, the reaction solvents were removed under reduced pressure to give crude *tert*-butyl 3,5-dimethylpiperazine-1-carboxylate (Compound **7**) as a yellow oil (1.8 g, Y = 96%), which was used in the next step without purification. LC-MS (ESI): *m*/*z* (M + 1)^+^ = 215.10.

General Procedure for the Preparation of Compound **8**

**Scheme 8:**
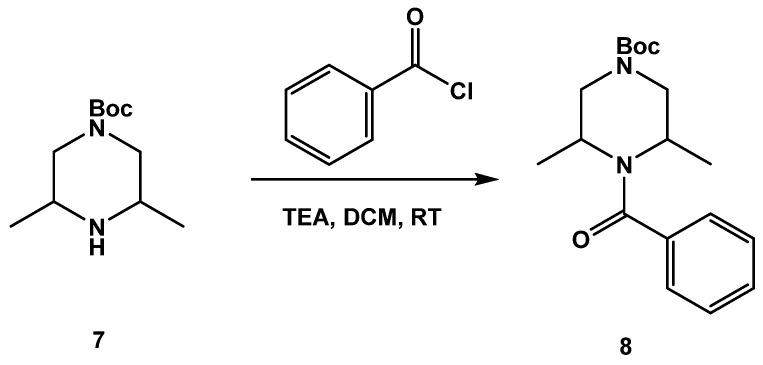
Preparation of Compound **8**.

To a mixture of Compound **7** (1.0 g, 4.7 mmol) in DCM (10 mL) was added benzoyl chloride (787.0 mg, 5.6 mmol) and TEA (944.0 mg, 9.3 mmol) at RT (Scheme 8). After stirred at RT for 1 h, the reaction mixture was diluted with DCM (10 mL) and washed with brine (10 mL × 3). The organic layer was dried over anhydrous Na_2_SO_4_, filtered, and concentrated under reduced pressure. The residue was purified by silica gel column chromatography (PE/EA = 3:1, *v*/*v*) to give tert-butyl 4-benzoyl-3,5-dimethylpiperazine-1-carboxylate (Compound **8**) as brown solid (1.4 g, Y = 94.0%). LC-MS (ESI): *m*/*z* (M + 1)^+^ = 319.17.

General Procedure for the Preparation of Compound **9**

**Scheme 9:**
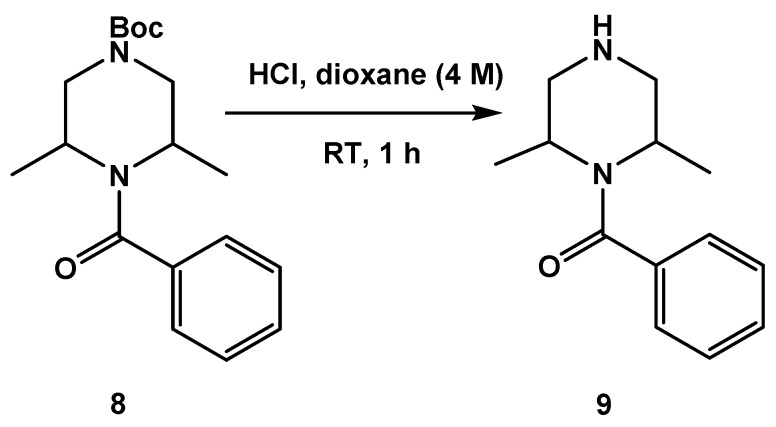
Preparation of Compound **9**.

A mixture of Compound **8** (1.0 g, 8.8 mmol) in HCl/dioxane (4 M, 10 mL) was stirred at RT for 1h (Scheme 9). The reaction solvents were removed in vacuo to give crude (2, 6-dimethylpiperazin-1-yl)(phenyl) methanone (Compound **9**) as a brown solid (1.1 g), which was used in the next step without purification. LC-MS (ESI): *m*/*z* (M + 1)^+^ = 219.39.

General Procedure for the Preparation of Compound **SC54**

**Scheme 10:**
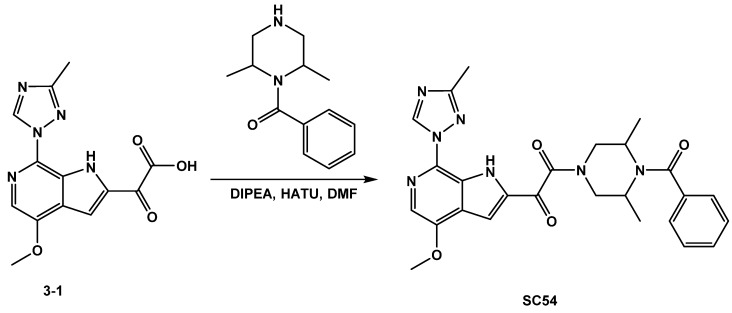
Preparation of Compound **SC54**.

To a solution of Compound **3-1** (28.0 mg, 0.09 mmol) in DMF (1 mL) was added (2,6-dimethylpiperazin-1-yl)(phenyl) methanone (Compound **9**, 24.0 mg, 0.11 mmol), HATU (42.4 mg, 0.11 mmol), and DIPEA (24.0 mg, 0.19 mmol) at 0 °C (Scheme 10). After the reaction mixture was stirred at RT for 1 h, the reaction mixture was diluted with ethyl acetate (20 mL) and washed with brine. The organic layer was dried over anhydrous Na_2_SO_4_, filtered, and concentrated under reduced pressure. The residue was purified by prep-HPLC (C18, 40–100% MeCN in H_2_O with 0.1% formic acid) to give 1-(4-benzoyl-3,5-dimethylpiperazin-1-yl)-2-(4-methoxy-7-(3-methyl-1H-1,2,4-triazol-1-yl)-1H-pyrrolo[2,3-c]pyridin-3-yl)ethane-1,2-dione as white solid (Compound **SC54**, 11.0 mg, Y = 24.0%). LC-MS (ESI): *m*/*z* (M + 1)^+^ = 502.22; ^1^H-NMR (400 MHz, CDCl_3_) δ 11.04 (br. s, 1H), 9.11 (s, 1H), 8.25 (d, *J* = 3.2 Hz, 1H), 7.77 (s, 1H), 7.44–7.42 (m, 3H), 7.38–7.36 (m, 2H), 4.49–4.46 (m, 3H), 4.06 (s, 3H), 3.78–3.75 (m, 1H), 3.41–3.37 (m, 1H), 3.09–3.05 (m, 1H), 2.56 (s, 3H), 1.42–1.39 (m, 6H).

### 3.2. Cells

HEK293T cells (gift from Dr. Irwin Chaiken, Drexel University, Philadelphia, PA, USA) were cultured in Dulbecco’s Modified Eagle’s Medium (DMEM). The medium was supplemented with 10% FBS, 100 U/mL penicillin, 100 µg/mL streptomycin, and 2 mM l-glutamine. Human astroglioma U87 cells (obtained from Prof. Hongkui Deng, Peking University, and Prof. Dan Littman, New York University, USA, through the AIDS Research and Reference Reagent Program, Division of AIDS, NIAID, NIH) [15,16] express CD4 and CCR5 receptors. U87 cells were cultured in DMEM, supplemented with 10% FBS, 100 U/mL penicillin, 100 µg/mL streptomycin, 2 mM l-glutamine, 300 µg/mL G418 (Thermo Scientific, Waltham, MA, USA), and 1 µg/mL Puromycin (Thermo Scientific). Both cell lines were incubated continuously at 37 °C, ensuring a humidified 5% CO_2_ environment.

### 3.3. Proteins

The recombinant, truncated trimer B41 SOSIP.664 gp140 was expressed in HEK293F cells. Post-expression, the soluble trimer was purified by mAb 2G12-affinity chromatography followed by size-exclusion chromatography as described previously [6]. Through NIH AIDS Reagent Program, Division of AIDS, NIAID, NIH, the recombinant human antibody IgG b12 anti-HIV-1 gp120 was acquired. The monoclonal form of the anti-HIV-1 gp120 antibody, IgG1 b12, was obtained from Dr. Dennis Burton and Carlos Barbas. As previously described, p24 was produced in-house [17]. A vector containing His-tagged HIV-1_NL4-3_CA on the C-terminus (a gift from Dr. Eric Barklis, Oregon Health and Science University, Portland, OR, USA) was transformed into BL21-Codon Plus (DE3)-RIL Competent Cells (Agilent Technologies, Wilmington, DE, USA). The vector expressed in autoinduction ZYP-5052 medium at 30 °C, and remained shaking overnight at 250 rpm [18]. Bacterial cultures were spun down at 8000 rpm. After discarding the supernatant, the remaining pellet was resuspended using 1 × PBS. The resuspension was lysed by sonication and spun down at 45,000 rpm. The supernatant was filtered through a 0.45 µm filter, and promptly applied to a Talon cobalt resin affinity column (Clonetech Laboratories, Mountain View, CA, USA). Elution of bound protein was accomplished using 1 × PBS with 250 mM imidazole. The elutions comprising of purified CA-H6 were merged and dialyzed overnight into 20 mM Tris-HCl pH 8.0. Subsequently, these were concentrated to 120 µM, flash-frozen, and stored at −80 °C.

### 3.4. Production of Pseudotyped Viruses

HEK293T cells were plated in 6-well plates with density of 1 × 10^6^ cells/well. After 24 h, the two vectors (vector 1 and vector 2) were co-transfected in a 3:4 ratio, respectively [16]. Vector 1 is an HIV-1 pNL4-3-Luc+R-E plasmid with two main features. The vector contains a premature stop codon in the Env gene and replaces HIV-1 Nef gene with the luciferase-reporter gene [19]. Vector 2 is a plasmid expressing the HIV-1_B41_ gp160 Env [20]. Transient transfections of these vectors were carried out via calcium phosphate (ProFection Mammalian Transfection System, Promega, Madison, WI) for 5 h in DMEM without supplements. After the 5 h incubation period, the medium was replaced with fresh culture media. Seventy-two hours post-transfection, virus was collected by filtering supernatants. The result was single-round infectious virus with pseudotyped envelopes that contained a luciferase reporter [16]. This was aliquoted and stored at −80 °C.

### 3.5. ELISA-Based Quantification of p24 Content

Mouse anti-p24 (Abcam, ab9071) coated an ELISA plate (50 ng/well) overnight at 4 °C. Following the overnight incubation, the plate was blocked for 2 h with 3% (*w*/*v*) room temperature BSA and washed with 0.5% (*v*/*v*) Tween in PBS. Pseudoviral stocks were lysed using 0.1% (*v*/*v*) Triton X-100 (Sigma-Aldrich, St. Louis, MO, USA) at 37 °C for 1 h and added to the plate overnight at 4 °C. Simultaneously, p24 protein (expressed and purified as previously described) was used as a standard. The next day, 0.5% PBST was used to wash the plate. Subsequently, rabbit anti-p24 (Abcam, ab63913) was added in a 1:5000 dilution at room temperature. After 2 h, PBST was used to wash unbound rabbit anti-p24. Next, goat anti-rabbit-HRP was added in a 1:5000 dilution at room temperature for one hour. PBST was used to thoroughly wash the plate. Subsequently, a solution of 0.4 mg/mL *o*-phenylenediamine in a phosphate-citrate buffer with sodium perborate (Sigma-Aldrich) was added. This was incubated in the dark for 30 min. Using Multiskan™ GO Microplate Spectrophotometer (Thermo Scientific), the optical densities were measured at 450 nm.

### 3.6. Single-Round Infection Assay

The single-round HIV-1 infection assay was performed as previously described [19,21,22]. U87.CD4.CCR5 cells were seeded in Greiner Bio-one 96-well luminometer-compatible tissue culture plates with a density of 1.2 × 10^4^ cells/well. After 24 h, compounds underwent serial dilutions in DMEM. DMSO was used as a vehicle control (Sigma). These dilutions were mixed with thawed pseudotyped HIV-1 virus (normalized to p24 content) and added to the U87 cells 100 µL/well. The plates were incubated for 48 h at 37 °C. Post-incubation, the medium was aspirated from each well, and 50 μL/well of 1X luciferase lysis buffer (Promega) was added. The plates were placed at −80 °C for one freeze-thaw cycle. After thawing, 50 µL/well of the luciferase assay substrate (Promega) was added and the luciferase activity of each well was measured by a GloMax 96 microplate luminometer (Promega).

### 3.7. SPR Direct Interaction Analysis

#### 3.7.1. Immobilization of Env Constructs

A ProteOn XPR36 SPR Protein Interaction Array System (Bio-Rad Laboratories, Hercules, CA, USA) was used to analyze kinetic interactions of the compounds to the B41 SOSIP.664 gp140 at 25  °C. First, the ProteOn GLH sensor chip was preconditioned. This was done with two 10 second pulses of the following: 50 mM NaOH, 100 mM HCl, and 0.5% (*w*/*v*) sodium dodecyl sulfide. PBST buffer (20 mM Na-phosphate, 150 mM NaCl, and 0.005% (*v*/*v*) polysorbate 20, pH 7.4) was then used to equilibrate the system. The activation of the GLH sensor chip surface occurred by a 10 minute injection with a 1:100 dilution of a 1:1 mixture of 1-ethyl-3-(3-dimethylaminopropyl)carbodiimide hydrochloride (0.2 M) and sulfo-*N*-hydroxysuccinimide (0.05 M). Promptly following chip activation, 100 µg/mL soluble gp140 B41.SOSIP.664 trimers in 10 mM sodium acetate (pH 4.5) were injected across ligand flow channels for 15 min at a flow rate of 25 µL/min. After immobilization of the soluble cleaved construct, excessive active ester groups were capped by a 5 min injection of 1 M ethanolamine HCl at a pH of 8.5. This resulted in immobilized Env constructs at a density of 14,000 RUs (response unit, which is an arbitrary unit that corresponds to 1 pg/mm^2^). As a control, IgG b12 in 10 mM sodium acetate (pH 4.5) was used as a reference surface and matched the density of the immobilized SOSIP.

#### 3.7.2. Direct Binding Analysis

The volume of compound stock solutions was increased to 30 μL with 100% DMSO. PBST pH 7.4 was added to the compound stocks to reach a final volume of 1 mL. This is required in order to match the concentration of DMSO of the compound stocks to that of running buffer (3% DMSO). As stated in the results, the starting concentrations of the serial dilutions were then prepared in running buffer (PBS, 3% (*v*/*v*) DMSO, 0.005% (*v*/*v*) polysorbate 20, pH 7.4). These dilutions were injected to flow across the surfaces at a rate of 100 μl min^−1^, for a 2-min association phase, followed by up to a 10-min dissociation phase. The Proteon instrument’s "one shot kinetics" completed these phases with ease [23]. Data were analyzed using the ProteOn Manager Software version 3.0 (Bio-Rad). The responses of a buffer injection and from the reference flow cell were subtracted to account for non-specific binding and injection artifacts. Experimental data were fitted to a simple 1:1 binding model. The average association [k_a_] and dissociation [k_d_] rates generated from 3–5 data sets were averaged and defined the equilibrium dissociation constant (K_D_).

### 3.8. Metabolic Stability Evaluation of Compounds in Human Liver Microsomes.

Metabolic stability analysis was performed as outlined in Lu et al. [14].

Materials: Warfarin was purchased from Sigma (St. Louis, MO, USA). Testosterone was purchased from Acros (Geel, Belgium). Propranolol was purchased from TRC (Toronto, Ont., Canada). NADPH was purchased from Roche (Shanghai, China); all inorganic salts erre of analytical grade and were purchased from Sinopharm Chemical Reagent Co. (Shanghai, China). All organic solvents were of HPLC grade and were purchased from Sigma-Aldrich, USA. Pooled human liver microsomes (HLM, Batch # 452161) were purchased from BD Gentest Corporation (Woburn, MA, USA). Distilled water, prepared from demineralized water, was used throughout the study.

Procedure:

Preparation of solutions. Test compound was weighed and dissolved in 100% DMSO to get 10 mM stock solution. The stock solution was diluted to 100 μM with mixture of acetonitrile and H_2_O (1:1). The final concentrations of DMSO and acetonitrile were equal to or less than 0.1%. Stock solutions of testosterone, warfarin, and propranolol were prepared to concentrations of 10 mM in 100% DMSO, respectively. The stock solution for each compound was diluted into 100 μM with mixture of acetonitrile and H_2_O (1:1). The final concentrations of DMSO and acetonitrile were equal or less than 0.1%.

Microsomal incubation. Liver microsomes incubations were conducted in duplicate in 96-well plates. Each well contained 40 µL of 0.1M potassium phosphate buffer (pH 7.4), 4.125 mM MgCl2, 0.625 mg/mL human liver microsomes, and test compound (1.25 µM) or positive control. After a 5-min preincubation at 37 °C, 10 µL of 5.0 mM NADPH in 0.1M potassium phosphate buffer was added to initiate the enzymatic reaction. The final component concentrations were 0.1 M potassium phosphate buffer (pH 7.4); 1.0 mM NADPH; 3.3 mM MgCl2; 0.5 mg/mL human liver microsomes and test compound (1.0 µM) or positive control (1.0 µM). Reactions were terminated at various time points (0, 5, 10, 20, 40 min) by adding 150 µL of ice-cold methanol and acetonitrile (1:1, *v*/*v*) containing the internal standard. A parallel incubation was performed using 0.1 M potassium phosphate buffer (pH 7.4) instead of NADPH as the negative control and reactions was terminated at 40 min after incubation at 37 °C.

LC-MS/MS analysis: A Waters liquid chromatographic system was used. Chromatographic separation of compounds was achieved using various ratios of buffer A (water, 0.1% formic acid) and buffer B (acetonitrile, 0.1% formic acid) and an ACQUITY UPLC BEH C18 column (50 × 2.1 mm, 1.7 µm) at ambient temperature (25 °C). The flow rate was maintained at 0.6 mL/min. Detection was performed on an API4000 Q-Trap mass spectrometer equipped with TurboIonSpray (ESI) Interface (Applied Biosystems, Concord, Ontario, Canada). Analyst 1.5 software packages (Applied Biosystems) were used to control the LC-MS/MS system, and for data acquisition and processing. 

Data analysis: The peak area ratio of a test compound to internal standard was plotted as a percentage of the relevant zero time point control (%remaining) for each reaction. The rate of metabolism (k) is the slope of the linear regression from log percentage remaining versus incubation time. The in vitro T½ was calculated as −0.693/k. The calculated rate was used to extrapolate the in vivo parameters: Intrinsic clearance (Clint) = k/c, where c is the microsomal protein concentration (0.5 mg/mL for liver microsomes); for a human, apparent clearance (Clapp) = Clint × a × b/d (mL/min/kg), where a, b, and d are the scaling factors for normalizing Clint to human body weight. The scaling factors a = 45 mg/g (microsomal protein/liver weight), b = 1500 g (liver weight), and d = 70 kg (body weight); hepatic clearance (Clh) = (Clapp × Q)/Clapp + Q), where Q = 20 mL/min/kg (liver blood flow); hepatic extraction ratio (Eh) = (Clh/Q) × 100 (%).

## 4. Conclusions

In this short communication, we applied several computational analyses to ascertain whether or not we could predict the metabolic stabilities of our first-generation scaffold-hopped entry inhibitors. We computationally determined drug-like properties, predicted the major metabolizing cytochrome P450 isoform, assesses metabolic liability, and performed affinity predictions of the compounds with CYP3A4. This combined analysis suggested that compounds **SC28** and **SC46** may have increased metabolic stability compared to the parental molecule temsavir/BMS-626529. This prediction was borne out by experimental determination of the compound half-lives in human liver microsome stability assays. Excitingly, **SC28** had more than twice the metabolic stability of temsavir/BMS-626529, and its increase even over **SC46** demonstrates that the orientation of the core replacement is critical for this increase, probably due to its effect on the affinity of the compound for binding to CYP3A4. Moreover, both **SC28** and **SC46** had very low toxicities, with therapeutic indices (TI: CC_50_/IC_50_) of over 10,000 (see supplementary information). This work also establishes another addition to our computational workflow that can be used to predict the metabolic stability of next-gen compounds, thus further streamlining future design processes.

## Figures and Tables

**Table 1:** Composite site lability (CSL) scores, number of labile sites, and predicted CYP3A4 affinities of compounds used in this study. CSL values and the number of labile sites were determined using StarDrop (version 6.6). Affinity to CYP3A4 was predicted using SeeSAR (version 9.2).

Compound	CSL 3A4	Labile Sites	Predicted 3A4 Affinity (M)
SC11	0.9514	3	0.004849324
SC12	0.9627	4	0.083703353
SC15	0.9564	3	0.003455543
SC28	0.9516	2	0.767515588
SC46	0.9474	3	0.166818977
SC54	0.9396	3	0.006416669
BMS-626529	0.9416	3	0.000802

**Table 2:** Metabolic stabilities of control and test compounds in a human liver microsome assay. Predictive PK parameters were derived as outlined in Materials and Methods.

Compound ID	k	T1/2	Clint	Clapp	Clh	Eh
		(min)	(mL/min/mg)	(mL/min/kg)	(mL/min/kg)	(%)
Testosterone	0.05134	13.5	0.1027	99.005	16.639	83.19
Propranolol	0.01305	53.1	0.0261	25.170	11.145	55.72
Warfarin	0.00352	196.6	0.0070	6.797	5.073	25.37
SC11	0.07253	9.6	0.1451	139.887	17.498	87.49
SC12	0.105828	6.548348	0.211656436	204.0972779	18.21506087	91.0753
SC15	0.05268	13.2	0.1054	101.588	16.710	83.55
SC28	0.00479	144.6	0.0096	9.245	6.322	31.61
SC46	0.00760	91.2	0.0152	14.657	8.455	42.28
SC54	0.022234	31.16817	0.04446845	42.8802911	13.63870629	68.19353
BMS-626529	0.010075	68.78436	0.020149929	19.43028912	9.855514403	49.27757

## Supplementary Materials

The supplementary materials are available online.
