# Supplementary material for: Composition and Orientation of the Core Region of Novel HIV-1 Entry Inhibitors Influences Metabolic Stability
Source: Molecules. 2020 Mar 21;25(6):1430. doi: 10.3390/molecules25061430 (PMC7144373; doi:10.3390/molecules25061430)
Supplement: Supplementary file 1 [file molecules-25-01430-s001.zip › molecules-746319-supplementary.pdf]

Supplemental Methods

Cellular Toxicity

The toxicity of compounds was determined by testing the viability of U87.CD4.CCR5 cells using the cell proliferation and cytotoxicity assay (Dojindo Molecular Technologies, Rockville, MD, USA). Per the manufacturer's instructions, cells were seeded in a 96-well tissue culture plate (Olympus plastics, San Diego, CA, USA) at a density of 1.2×10^4 cells/well. After 24 h, cells were treated with compounds at a starting concentration of 1mM with 1:5 serial dilutions (or DMSO control). After a 48 h incubation at 37 °C, 10 µL of the CCK-8 solution was added to each well, and the plate was incubated for 4 h at 37 °C. Subsequently, the absorbance of each well was measured in a Multiskan™ GO Microplate Spectrophotometer (Thermo Scientific). Untreated cells were used as a background control, and 0.1% SDS treated wells were used as a positive control. The half-maximal inhibitory concentration (CC_{50}) was determined by fitting datapoints with a logistic curve fit to determine the concentration at which there was 50% cell viability of treated cells in comparison with the control cells.

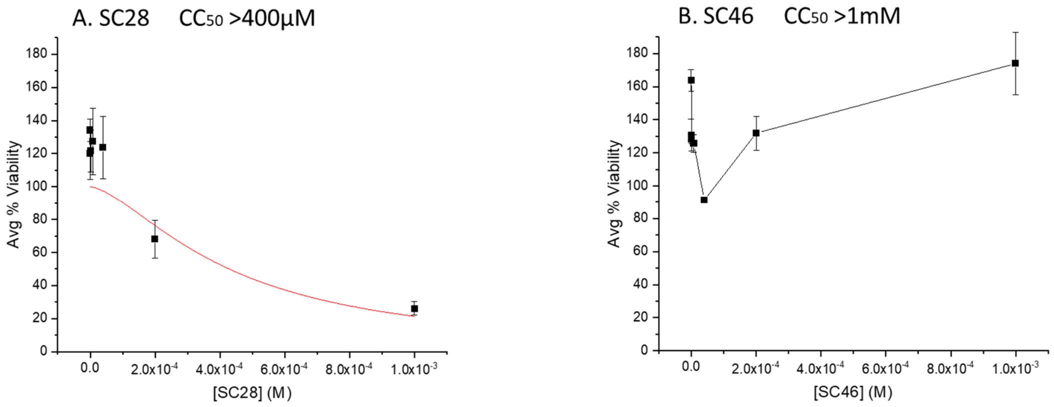

Figure S1. Toxicity of compounds (**A**) **SC28** and (**B**) **SC46** starting at 1mM with 1:5 dilutions. The cell viability assay was performed with U87.CD4.CCR5 cells. The red line represents the logistic fit of the data points from which the CC_{50} was derived. Error bars represent the standard deviations.

Table 1. Potency, toxicity, and therapeutic index of compounds **SC28** and **SC46**.

Compound	HIV-1$_{B41}$ IC_{50}	CC_{50}	Therapeutic Index (CC_{50}/IC_{50})
SC28	35 nM	>0.4 mM	>11428.57
SC46	91.5 nM	>1 mM	>10989.01
